# Assessment of the relationship between awareness of popular diets and body image among young women: a cross-sectional study

**DOI:** 10.1186/s41043-026-01388-6

**Published:** 2026-07-07

**Authors:** Ayşe Nur Kahve, Ebru Bayrak

**Affiliations:** 1 Department of Sports and Health, Aksaray University, Aksaray, Türkiye; 2 Nutrition and Dietetics, Selçuk University, Konya, Türkiye

**Keywords:** Body image, Diet awareness, Eating habits, Female health, Popular diets

## Abstract

**Background:**

Body image dissatisfaction is common among young women and has been associated with various health-related behaviors. Popular diets, often promoted through media and social platforms, are widely recognized among university students; however, evidence regarding the relationship between body image perception and awareness or use of such diets remains limited.

**Methods:**

This cross-sectional study included 580 female university students aged 18–33 years in Türkiye. Data were collected using a sociodemographic questionnaire, a food frequency questionnaire, and the Body Image Scale (BIS). Awareness, perceived healthiness, and practices of popular diets were assessed. Statistical analyses included chi-square tests, analysis of variance, correlation analyses, and effect size measures (Cramér’s V and eta-squared).

**Results:**

Lower BIS scores were associated with higher BMI, more frequent meal skipping, and lower water intake (*p* < 0.05). Students with lower body image scores also reported a higher prevalence of chronic diseases. Awareness and practices of most popular diets, including ketogenic, Mediterranean, and gluten-free diets, did not differ significantly according to body image level. However, after Bonferroni correction (*p* < 0.016), none of the diet-related comparisons remained significant; only nominal differences were observed for detox diet practice (*p* = 0.029) and awareness of monotrophic diets (*p* = 0.043), with small effect sizes.

**Conclusions:**

Body image perception among young women appears to be related to certain health behaviors but shows limited association with awareness or practices related to most popular diets. These findings suggest that body image alone may not be a primary determinant of dietary trends among university students. Interventions addressing body image should consider broader social, cultural, and behavioral factors to promote healthy eating behaviors among young women.

**Supplementary Information:**

The online version contains supplementary material available at https://doi.org/10.1186/s41043-026-01388-6.

## Background

Body image refers to an individual’s perceptions, thoughts, feelings, and attitudes toward their own body and physical appearance [1]. It is a multidimensional construct shaped by biological, psychological, social, and cultural factors, and may influence health-related attitudes and behaviors [2]. A discrepancy between an individual’s perceived body shape and the culturally idealized body standard is a significant risk factor for body dissatisfaction [3]. Such a discrepancy can negatively impact an individual’s lifelong health behaviors [4].

Body image is associated with numerous factors, including gender, age, environmental conditions, cultural influences, and personal history [5, 6]. Studies have reported that body image concerns and body dissatisfaction are higher in women than in men [7–9]. Additionally, studies among university students show that female students strive for weight loss more than male students, and that this behavior is almost considered normal for female students [10, 11]. Turkish university students indicate similar trends, showing that female students often report higher body dissatisfaction and stronger desire for weight loss compared to their male peers [12, 13]. Cultural norms in Turkey, influenced by social media and societal beauty ideals, may further contribute to these perceptions and related dietary behaviors [14]. University students tend to have higher levels of body image anxiety compared to other age groups [15, 16]. Students majoring in health care pay more attention to their individual health, tend to adopt healthier lifestyles, and have more positive perceptions of their health and bodies compared to students in other academic disciplines [17, 18]. Negative body image is a public health concern due to its association with disordered eating behaviors such as overeating and unhealthy weight control [19, 20] and its negative health effects [21–23].

Given the established relationship between body image perception and health-related behaviors, interest in dieting practices and popular dietary trends has become increasingly prominent, particularly among young women concerned with weight and appearance. Dieting to achieve an ideal body weight is common among university students [24]. Popular diets marketed as “healthy” solutions have increasingly blurred the distinction between evidence-based nutrition and restrictive dietary practices, particularly among young women [25]. Some popular diets are characterized by restrictive dietary patterns, which may increase the risk of inadequate nutrient intake and reinforce socially idealized body standards, particularly among young women [26]. These diets include intermittent fasting, monotrophic diets, alkaline diets, Karatay diets, blood type diets, and gluten-free diets [27]. Many are marketed as promoting health but exploit aesthetic concerns for commercial gain and lack scientific evidence [28]. Accurate weight perception relates to healthier eating [29, 30], while body dissatisfaction is linked to higher dieting interest and thin-ideal endorsement [31], potentially harming health behaviors [32–34].

Although previous studies have extensively examined the relationship between body image dissatisfaction and disordered eating behaviors, limited research has specifically explored how body image perception relates to awareness, perceived healthiness, and actual practices regarding popular diets among young women. Moreover, most existing studies have focused primarily on eating pathology or weight-control behaviors rather than broader perceptions and engagement with contemporary popular dietary trends. Therefore, this study aimed to examine the association between body image perception and health-related dietary beliefs, behaviors, and perceptions regarding popular diets among female university students in Türkiye.

## Methods

### Participants and study design

This cross-sectional study included 580 female students enrolled in the Departments of Nutrition and Dietetics, Midwifery, Child Development, Health Management, and Social Work at the Faculty of Health Sciences of Selçuk University, located in Konya, Turkey. Sample size estimation was also considered in relation to the primary categorical analyses conducted using chi-square tests. Based on Cohen’s guidelines for chi-square tests, detecting a small effect size (w = 0.10) at α = 0.05 with 80% power would require approximately 785 participants, whereas detecting a small-to-moderate effect size (w = 0.15) would require approximately 349 participants. Given that most expected associations were anticipated to be small, the achieved sample size (*n* = 580) was considered likely sufficient to detect small-to-moderate effect sizes in categorical comparisons between body image groups, although very small associations may still have gone undetected. Data collection was conducted between February and June 2020, partially overlapping with the early phase of the COVID-19 pandemic in Türkiye. The inclusion criteria for the students were voluntary participation in the study, absence of communication problems that could interfere with answering the questionnaire, and no self-reported history of a previously diagnosed psychological disorder at the time of data collection. Participants who did not meet these criteria were excluded from the study.

## Data collection tools

### Questionnaire design

In the first part of the study, a questionnaire developed by the researchers based on a literature review was used to collect basic information on university students’ age, height, weight, income status, body mass index, health status, chronic diseases, and dietary habits. The researcher developed questionnaire was prepared based on previous studies examining body image, dieting behaviors, and awareness and practices related to popular diets among university students [14, 23, 35]. The questionnaire included items related to sociodemographic characteristics, dietary habits, awareness of popular diets, previous diet practices, and perceptions regarding the healthiness of these diets. Before the main data collection, the questionnaire was pilot-tested with 25 female university students to evaluate clarity, comprehensibility, and applicability, and minor wording revisions were made accordingly.

Awareness was assessed with the question: “Have you heard of the following diet?” (Yes/No).

Practice was assessed with: “Have you ever practiced this diet?” (Yes/No).

Perceived healthiness was assessed with: “Do you consider this diet to be healthy?” (Yes/No). In addition, a food frequency questionnaire was used to assess students’ food preferences [36].

### Body image scale (BIS)

Developed by Secord and Jourard [37] to assess individuals’ satisfaction with their own body parts and functions, the BIS scale was adapted into Turkish by Hovardaoğlu [38] and is a unidimensional measurement tool consisting of 40 items, each of which describes a body part or organ (such as arms, legs, or face) or a function (such as sexual activity level). Items are scored on a five-point Likert-type scale as follows: “5 - Very much liked,” “4 - Liked,” “3 - Neutral,” “2 - Disliked,” and “1 - Very much disliked.” The items assess the level of satisfaction, with a minimum score of 1 and a maximum score of 5. The Body Image Scale used in this study corresponds to the Turkish adaptation of the Body Cathexis Scale originally developed by Secord and Jourard [37]. On the BIS scale, a score between 40 and 135 indicates a “low” body image level, and a score between 136 and 200 indicates a “high” body image level. This categorization was performed according to the cutoffs suggested by Hovardaoğlu [38], which have been used in prior Turkish studies. In line with previous Turkish studies using the Body Image Scale, categorized body image groups (low/high) were used to facilitate group-based comparisons in categorical analyses and to improve comparability with the existing national literature. The total score ranges from 40 to 200, with higher scores indicating greater satisfaction with one’s body image. Hovardaoğlu [38] reported a Cronbach’s alpha coefficient of 0.91 for the entire scale. In this study, the Cronbach’s alpha coefficient was calculated as 0.905, demonstrating excellent reliability for the scale.

### Statistical analysis

SPSS 22.0 was used for statistical analyses. Normality was assessed using skewness and kurtosis values within ± 2.0 [39]. Additionally, the Kolmogorov–Smirnov Z test was applied, and p values greater than 0.05 indicated normal distribution. In addition, histograms and Q–Q plots were visually inspected to further evaluate the distribution of the data. Given the large sample size (*n* = 580), slight deviations from normality were considered acceptable in accordance with the Central Limit Theorem. The participants’ sociodemographic characteristics and BIS mean scores are given as mean, standard deviation and percentage distribution. The relationship between sociodemographic characteristics and BIS was analyzed with One-way ANOVA. The relationship between women’s hearing about, applying and finding popular diets healthy and the BIS was determined using the chi-square test. Cramér’s V test was used to determine the strength of the association (effect size) between the variables following the chi-square test. A value of V = 0.1 was interpreted as a small effect, V = 0.3 as a medium effect, and V = 0.5 as a large effect [40]. In addition, the eta squared (η²) test was used to evaluate the effect size following the analysis of variance. An eta squared value of η² = 0.01 indicates a small effect, η² = 0.06 a medium effect, η² = 0.14 a large effect, and η² = 0.20 a very large effect [41]. The study findings were analyzed at a 95% confidence interval and *p* < 0.05 significance level. Because three separate comparisons were conducted for each popular diet type (awareness, practice, and perceived healthiness), Bonferroni correction was applied within each diet category to reduce the risk of type I error associated with multiple testing. Accordingly, the adjusted significance threshold was set at *p* < 0.016 (0.05/3) for these comparisons. Nominal p-values are also reported for transparency. Additionally, a multivariable binary logistic regression analysis was performed as a supplementary analysis to explore factors independently associated with body image perception.

### Ethical considerations

The study was conducted following the Declaration of Helsinki Principles. To carry out the research, the approval of the Selçuk University Faculty of Health Sciences Ethics Committee for Non-Interventional Clinical Investigations was obtained (29.01.2020–137).

## Results

### Sample characteristics and scale scores

The mean age of the university students participating in the study was 20.7 ± 1.84 years (range: 18–33), and their mean body mass index (BMI) was 21.56 ± 4.69. Table 1 presents the comparisons between the descriptive characteristics of the students and the total scores of the scale. A statistically significant difference was found between students’ weights and their BIS scores (*p* = 0.011, *p* < 0.05). The effect size calculation revealed that the independent variable explained approximately 13.8% of the variance in body image (η²=0.138), which is considered a large effect.

**Table 1:** Relationship between some socio-demographic characteristics of female students and their body perception (*n* = 580)

Socio-demographic characteristics		BIS ($\bar{\rm X}$±SD)	f	*p* ^c^	Effect size (η²)	95% CI
**Age** ^**a**^	20.7 (1.84)	148.33 ± 23.85	1.187	0.289	0.024	
**Weight**,** kg**^**a**^	57.2 (8.95)		1.530	0.011*	0.138	
**Height**,** cm**^**a**^	163.2 (6.65)		1.214	0.198	0.066	
**Department** ^**b**^						
Nutrition and dietetics	138 (23.8)	145.3 ± 1.92	1.910	0.107	0.013	[141.52–149.14]
Midwifery	188 (32.4)	150.5 ± 1.74				[147.08–153.98]
Child development	55 (9.5)	146.7 ± 3.21				[140.27–153.15]
Health management	116 (20.0)	151.4 ± 2.38				[146.73–156.18]
Social work	83 (14.3)	145.0 ± 2.44				[140.18–149.92]
**Class** ^**b**^						
1 st year	143 (24.7)	147.6 ± 1.7	1.361	0.254	0.007	[144.22–150.98]
2nd year	156 (26.9)	148.4 ± 2.07				[144.35–152.53]
3rd year	137 (23.6)	145.7 ± 2.04				[141.73–149.80]
4th year	144 (24.8)	151.3 ± 2.04				[147.33–155.43]
**Monthly expenditure** ^**b**^						
0–4000 TL	261 (45.0)	149.5 ± 1.53	1.040	0.585	0.002	[146.58–152.60]
4000–8000 TL	269 (46.4)	146.9 ± 1.35				[144.32–149.65]
> 8000 TL	50 (8.6)	149.0 ± 3.91				[141.13–156.87]
**Monthly food expenditure** ^**b**^						
0–2000 TL	365 (62.9)	149.10 ± 1.29	1.076	0.342	0.003	[146.57–151.64]
2000–4000 TL	183 (31.6)	147.74 ± 1.63				[144.52–150.95]
> 4000 TL	32 (5.5)	142.91 ± 4.32				[134.09–151.72]

^a^ Data presented as mean (SD), ^b^Data presented as n (% of sample), ^c^One-Way ANOVA, *p < 0.05

### Participants’ health status and nutritional habits

Regarding the presence of chronic diseases, 124 participants (21.4%) reported having at least one chronic disease. There was no statistically significant association between body image group and the overall presence of chronic disease. However, when specific chronic disease types were analyzed separately, a significant difference emerged between the groups (χ²(9) = 18.062, *p* = 0.034, Cramér’s V = 0.382). Among chronic conditions, anemia was the most frequently reported (8.8%), followed by gastritis-ulcer (4.5%) and thyroid disorders (2.8%) (Table 2).

**Table 2:** The relationship between female students’ health status, nutritional habits and body perception

Variables	Total	Low body image (*n* = 176)	High body image (*n* = 404)	χ² value	df	*p*	Effect size Cramer’s V
**Chronic diseases** ^**a**^							
Yes	124 (21.4)	46 (7.9)	78 (13.4)		-	0.01* ^c^	
No	456 (78.6)	130 (22.4)	326 (56.2)				
**Distribution of chronic diseases** ^**a**^							
Diabetes	3 (0.5)	2 (0.3)	1 (0.2)	18.062	9	0.034* ^**b**^	0.382
Anemia	51 (8.8)	26 (4.5)	25 (4.3)				
Constipation	8 (1.4)	2 (0.3)	6 (1.1)				
Gastritis-Ulcer	26 (4.5)	6 (1.1)	20 (3.4)				
Hypertension	3 (0.5)	1 (0.2)	2 (0.3)				
Cholesterol	1 (0.2)	1 (0.2)	-				
Kidney	8 (1.4)	3 (0.5)	5 (0.9)				
Thyroid	16 (2.8)	1 (0.2)	15 (2.6)				
Joint and bone diseases	7 (1.2)	4 (0.7)	3 (0.5)				
Cardiovascular diseases	1 (0.2)	-	1 (0.2)				
**Main meals/day** ^**a**^							
1	28 (4.8)	11 (1.9)	17 (2.9)		-	0.226^c^	
2	308 (53.1)	98 (16.9)	209 (36.0)				
3	242 (41.7)	66 (11.4)	176 (30.3)				
≥4	2 (0.4)	1 (0.2)	1 (0.2)				
**Snacks/day** ^**a**^							
No	134 (23.1)	35 (6.0)	99 (17.1)			0.732^c^	
1	225 (38.8)	77 (13.2)	148 (25.5)				
2	173 (29.8)	51 (8.7)	122 (21.0)				
3	39 (6.7)	11 (1.9)	28 (4.9)				
≥4	9 (1.6)	2 (0.4)	7 (1.2)				
**Skipping meal** ^**a**^							
Yes	448 (77.2)	149 (25.7)	299 (51.6)	8.732	1	**0.003** ^b*^	0.123
No	132 (22.8)	27 (4.7)	105 (18.1)				
**Skippped meal** ^**a**^							
Breakfast	157 (27.1)	46 (7.9)	111 (19.1)		-	0.692^c^	
Afternoon	222 (38.3)	77 (13.3)	145 (25.0)				
Evening	14 (2.4)	5 (0.9)	9 (1.55)				
Snack	55 (9.4)	21 (3.6)	34 (5.9)				
**Reason for skipping meals** ^**a**^							
I don’t feel like it	179 (30.9)	69 (11.9)	110 (19.0)		-	0.226^c^	
I don’t have enough time	168 (29.0)	49 (8.4)	119 (20.5)				
For weight loss purposes	7 (1.2)	1 (0.2)	6 (1.1)				
None	95 (16.4)	31 (5.3)	64 (11.0)				
**Daily water consumption** ^**a**^							
0–1 L	228 (39.3)	79 (13.6)	149 (25.7)	3.156	2	0.206^b^	0.074
1–2 L	258 (44.5)	71 (12.2)	187 (32.2)				
≥ 2 L	94 (16.2)	26 (4.5)	68 (11.7)				
**Frequency of eating out** ^**a**^							
Every day	89 (15.3)	29 (5.0)	60 (10.3)	4.076	5	0.539 ^b^	0.084
2–3 days a week	266 (45.9)	81 (14.0)	185 (31.9)				
Once a week	90 (15.5)	21 (3.6)	69 (11.9)				
Once every 15 days	48 (8.3)	15 (2.6)	33 (5.7)				
Once a month	29 (5.0)	8 (1.4)	21 (3.6)				
Rarely	58 (10.0)	22 (3.8)	36 (6.2)				
**What does the popular diet mean to you?** ^**a**^							
Current diets	331 (57.0)	103 (17.8)	228 (39.3)	1.187	3	0.756 ^b^	0.045
Weight loss diets	96 (16.6)	27 (4.7)	69 (11.9)				
Diets beneficial to health	114 (19.7)	32 (5.5)	82 (14.1)				
Diets harmful to health	39 (6.7)	14 (2.4)	25 (4.3)				

^a^Data presented as n (% of sample), df= degrees of freedom, ^b^χ² test, ^c^Fisher’s Exact Test, *p < 0.05

No statistically significant differences were found between the body image groups in terms of the number of main meals consumed daily (*p* = 0.226), frequency of daily snacks (*p* = 0.732), daily water consumption (*p* = 0.206), or frequency of eating out (*p* = 0.539). A statistically significant relationship was found between meal skipping and body image levels (χ²=8.732, df = 1, *p* = 0.003, Cramér’s V = 0.123), indicating a small effect size. These findings suggest that students with lower body image perception were more likely to engage in meal skipping behavior (Table 2).

Correlation analysis revealed a significant negative relationship between BMI and body image scores (*r* =−0.082, *p* < 0.05), as well as between skipping meals and body image scores (*r*=−0.125, *p* < 0.01). These findings suggest that higher BMI and frequent meal skipping are associated with lower body image satisfaction. A significant positive correlation was found between daily water consumption and body image scores (*r* = 0.093, *p* < 0.05), indicating that greater water intake is linked to higher body image satisfaction. No significant correlations were observed between body image scores and age, monthly expenditure, food expenditure, number of main meals, or number of snacks (*p* > 0.05) (Table 3).

**Table 3:** Correlation between the total score of the BIS and certain parameters

Variables	*r*	95% CI	*p* value
**BMI**	−0.082	[−0.16, −0.01]	0.04
**Age**	0.024	[−0.06, 0.10]	0.61
**Monthly expenditure**	−0.034	[−0.11, 0.04]	0.39
**Monthly food expenditure**	−0.055	[−0.13, 0.02]	0.17
**Main meals/day**	0.062	[−0.02, 0.14]	0.14
**Snacks/day**	0.046	[−0.03, 0.12]	0.27
**Daily water consumption**	**0.093**	[0.01, 0.17]	0.02
**Skipping meals**	**−0.125**	[−0.20, −0.05]	0.003

*p < 0.05, **p < 0.01

An additional multivariable logistic regression analysis was conducted to further explore factors independently associated with body image perception. The results were generally consistent with the primary bivariate findings, with meal skipping and the presence of chronic disease remaining significantly associated with body image perception after adjustment for potential confounding variables. The findings are presented in Supplementary Table S1.

### Participants’ awareness of popular diets and body image perception

As presented in Table 4, the relationship between body image levels (low vs. high) and participants’ awareness, practice, and perceived healthiness of various popular diets was examined using chi-square tests. In general, no statistically significant differences were observed between the groups after Bonferroni correction. Nominal between-group differences were observed for awareness of the Monotrophic Diet (χ²=3.258, *p* = 0.043, Cramér’s V = 0.075; small effect) and practice of Detox Diets (χ²=4.752, *p* = 0.029, Cramér’s V = 0.091; small effect). However, these associations did not remain statistically significant after adjustment for multiple comparisons (*p* < 0.016), and no robust associations were observed between body image groups and popular diet awareness, practice, or perceived healthiness. Across all comparisons, Cramér’s V values were below 0.10, indicating weak associations.

**Table 4:** The relationship between awareness of popular diets and body image perception among female students

Variables	High body image (*n* = 404)		Low body image (*n* = 176)		χ² value	*p* ^a^	Effect size
	**Karatay Diet**		**Karatay Diet**				
	**Yes**	**No**	**Yes**	**No**			
**Diets you have heard of**	330 (81.7)	74 (18.3)	140 (79.5)	36 (20.5)	0.365	0.546	0.025
**Diets you have practiced**	18 (4.5)	386 (95.5)	7 (4.0)	169 (96.0)	0.068	0.794	0.011
**Diets you consider healthy**	44 (10.9)	360 (89.1)	14 (8.0)	162 (92.0)	1.175	0.278	0.045
	**Intermittent Fasting Diet**		**Intermittent Fasting Diet**				
	**Yes**	**No**	**Yes**	**No**			
**Diets you have heard of**	249 (61.6)	155 (38.4)	101 (57.4)	75 (42.6)	0.924	0.336	0.04
**Diets you have practiced**	23 (5.7)	381 (94.3)	8 (4.5)	168 (95.5)	0.319	0.572	0.23
**Diets you consider healthy**	78 (19.3)	326 (80.7)	35 (19.9)	141 (80.1)	0.026	0.871	0.007
	**Alkaline Diet**		**Alkaline Diet**				
	**Yes**	**No**	**Yes**	**No**			
**Diets you have heard of**	162 (40.1)	242 (59.9)	63 (35.8)	113 (64.2)	0.956	0.328	0.04
**Diets you have practiced**	5 (1.2)	399 (98.8)	1 (0.6)	175 (99.4)	0.537	0.464	0.03
**Diets you consider healthy**	16 (4.0)	387 (95.8)	2 (1.1)	174 (98.9)	3.706	0.157	0.08
	**Blood group Diet**		**Blood group Diet**				
	**Yes**	**No**	**Yes**	**No**			
**Diets you have heard of**	154 (38.1)	250 (61.9)	72 (40.9)	104 (59.1)	0.401	0.526	0.026
**Diets you have practiced**	6 (1.5)	398 (98.5)	1 (0.6)	175 (99.4)		0.681^b^	
**Diets you consider healthy**	19 (4.7)	385 (95.3)	7 (4.0)	169 (96.0)	0.151	0.698	0.016
	**Mediterranean Diet**		**Mediterranean Diet**				
	**Yes**	**No**	**Yes**	**No**			
**Diets you have heard of**	196 (48.5)	208 (51.5)	80 (45.5)	96 (54.5)	0.460	0.497	0.028
**Diets you have practiced**	21 (5.2)	383 (94.8)	9 (5.1)	167 (94.9)	0.002	0.966	0.002
**Diets you consider healthy**	75 (18.6)	329 (81.4)	27 (15.3)	149 (84.7)	0.879	0.349	0.039
	**Very low calorie Diet**		**Very low calorie Diet**				
	**Yes**	**No**	**Yes**	**No**			
**Diets you have heard of**	197 (48.8)	207 (51.2)	83 (47.2)	93 (52.8)	0.126	0.722	0.015
**Diets you have practiced**	6 (1.5)	398 (98.5)	7 (4.0)	169 (96.0)		0.072^b^	
**Diets you consider healthy**	4 (1.0)	400 (99.0)	1 (0.6)	175 (99.4)		1.000 ^b^	
	**DASH Diet**		**DASH Diet**				
	**Yes**	**No**	**Yes**	**No**			
**Diets you have heard of**	105 (26.0)	299 (74.0)	54 (30.7)	122 (69.3)	1.356	0.244	0.048
**Diets you have practiced**	20 (5.0)	384 (95.0)	-	176 (100.0)	0.874	0.350	0.039
**Diets you consider healthy**	168 (41.6)	236 (58.4)	9 (5.1)	167 (94.9)	0.007	0.934	0.003
	**Ketogenic Diet**		**Ketogenic Diet**				
	**Yes**	**No**	**Yes**	**No**			
**Diets you have heard of**	168 (41.6)	236 (58.4)	78 (44.3)	98 (55.7)	0.375	0.540	0.025
**Diets you have practiced**	6 (1.7)	397 (98.3)	1 (0.6)	175 (99.4)		0.673^b^	
**Diets you consider healthy**	18 (4.5)	386 (95.5)	7 (4.0)	169 (96.0)	0.068	0.794	0.011
	**Gluten-free Diet**		**Gluten-free Diet**				
	**Yes**	**No**	**Yes**	**No**			
**Diets you have heard of**	290 (71.8)	114 (28.2)	128 (72.7)	48 (27.3)	0.054	0.816	0.010
**Diets you have practiced**	10 (2.5)	394 (97.5)	8 (4.5)	168 (95.5)	1.747	0.186	0.055
**Diets you consider healthy**	75 (18.6)	329 (81.4)	27 (15.3)	149 (84.7)	0.755	0.385	0.036
	**Monotrophic diet**		**Monotrophic diet**				
	**Yes**	**No**	**Yes**	**No**			
**Diets you have heard of**	246 (60.9)	158 (39.1)	121 (68.8)	55 (31.3)	3.258	0.043*	0.075
**Diets you have practiced**	15 (3.7)	389 (96.3)	8 (4.5)	168 (95.5)	0.223	0.637	0.020
**Diets you consider healthy**	17 (4.2)	387 (95.8)	9 (5.1)	167 (94.9)	0.395	0.530	0.026
	**Detox Diet**		**Detox Diet**				
	**Yes**	**No**	**Yes**	**No**			
**Diets you have heard of**	313 (77.5)	91 (22.5)	140 (79.5)	36 (20.5)	0.307	0.579	0.023
**Diets you have practiced**	23 (5.7)	381 (94.3)	19 (10.8)	157 (89.2)	4.752	0.029*	0.091
**Diets you consider healthy**	62 (15.3)	342 (84.7)	27 (15.3)	149 (84.7)	0.000	0.999	0.000

*p < 0.05, Data presented as n (% of sample), ^a^χ² test, ^b^Fisher’s Exact Test

The relationship between the sources from which participants heard about popular diets and their body image levels was examined. The analysis revealed no statistically significant difference in the sources of information such as media, peers, or dietitians/health professionals based on body image levels (χ²(2) = 2.679, *p* = 0.262) (Table 5). This finding suggests that women, regardless of whether they have high or low body image, tend to hear about popular diets from similar sources.

**Table 5:** Resources informing female students about popular diets

Sources	High body image (*n* = 404)	Low body image (*n* = 176)	χ² value	df	*p* ^a^
**Media**	289	121	2.679	2	0.262
**Social environment**	66	38			
**Professional guidance (Dietitians and health workers)**	49	17			

^a^χ² test

### Food consumption frequencies of female students according to their body perception

The association between frequency of consumption of various food and body image perception was examined using chi-square tests. As shown in Table 6, no statistically significant differences were observed in the consumption frequencies of milk, yogurt/ayran, cheese, red meat, white meat, fish, eggs, legumes, fresh vegetables, fresh fruits, vegetable oils, solid fats, olive oil, bread, pasta/rice, tea, coffee, or soda between individuals with high and low body image perceptions (*p* > 0.05) (Table 6). The effect sizes, as indicated by Cramér’s V, were small across all comparisons, further supporting the lack of meaningful association.

**Table 6:** The relationship between food consumption frequency and body image perception among female students

Foods		Frequency of consumption						χ² value	df	*p* ^a^	Effect size Cramer’s V
		Daily	3–4 times per week	1–2 times per week	Every 15 days	Once a month	Do not consume				
		*n* (%)	*n* (%)	*n* (%)	*n* (%)	*n* (%)	*n* (%)				
Milk	High body image	46 (11.4)	83 (20.5)	105 (26.0)	62 (15.3)	54 (13.4)	54 (13.4)	4.741	5	0.448	0.090
	Low body image	14 (8.0)	33 (18.8)	60 (34.1)	24 (13.6)	23 (13.1)	22 (12.5)				
Yogurt/Ayran	High body image	89 (22.0)	175 (43.3)	102 (25.2)	24 (5.9)	8 (2.0)	6 (1.5)	3.170	5	0.674	0.074
	Low body image	44 (25.0)	73 (41.5)	39 (22.2)	15 (8.5)	4 (2.3)	1 (0.6)				
Cheese	High body image	191 (47.3)	132 (32.7)	52 (12.9)	12 (3.0)	5 (1.2)	12 (3.0)	2.416	5	0.789	0.065
	Low body image	77 (43.8)	60 (34.1)	26 (14.8)	3 (1.7)	4 (2.3)	6 (3.4)				
Red meat	High body image	5 (1.2)	78 (19.3)	157 (38.9)	94 (23.3)	45 (11.1)	25 (6.2)	4.513	5	0.478	0.088
	Low body image	1 (0.6)	29 (16.5)	66 (37.5)	43 (24.4)	29 (16.5)	8 (4.5)				
White meat	High body image	10 (2.5)	115 (28.5)	181 (44.8)	59 (14.6)	29 (7.2)	10 (2.5)	3.861	5	0.570	0.082
	Low body image	1 (0.6)	43 (24.4)	85 (48.3)	28 (15.9)	15 (8.5)	4 (2.3)				
Fish	High body image	1 (0.2)	6 (1.5)	54 (13.4)	88 (21.8)	202 (50.0)	53 (13.1)	4.757	5	0.446	0.091
	Low body image	-	1 (0.6)	20 (11.4)	30 (17.0)	103 (58.5)	22 (12.5)				
Egg	High body image	79 (19.6)	129 (31.9)	113 (28.0)	38 (9.4)	20 (5.0)	25 (6.2)	3.562	5	0.614	0.078
	Low body image	38 (21.6)	58 (33.0)	40 (22.7)	15 (8.5)	14 (8.0)	11 (6.3)				
Legume	High body image	1 (0.2)	20 (5.0)	117 (29.0)	178 (44.1)	53 (13.1)	19 (4.7)	3.884	5	0.692	0.082
	Low body image	5 (2.8)	48 (27.3)	90 (51.1)	20 (11.4)	8 (4.5)	5 (2.8)				
Fresh vegetables	High body image	70 (17.3)	180 (44.6)	113 (28.0)	26 (6.4)	11 (2.7)	4 (1.0)	5.122	5	0.401	0.094
	Low body image	29 (16.5)	70 (39.8)	50 (28.4)	16 (9.1)	10 (5.7)	1 (0.6)				
Fresh fruits	High body image	149 (36.9)	161 (39.9)	70 (17.3)	18 (4.5)	3 (0.7)	3 (0.7)	2.405	5	0.791	0.064
	Low body image	58 (33.0)	69 (39.2)	39 (22.2)	7 (4.0)	1 (0.6)	2 (1.1)				
Vegetable oils (corn oil, sunflower oil, hazelnut oil)	High body image	217 (53.7)	80 (19.8)	52 (12.9)	20 (5.0)	12 (3.0)	23 (5.7)	6.342	5	0.274	0.105
	Low body image	97 (55.1)	23 (13.1)	26 (14.8)	11 (6.3)	10 (5.7)	9 (5.1)				
Solid fats (butter, margarine, etc.)	High body image	79 (19.6)	96 (23.8)	112 (27.7)	44 (10.9)	40 (9.9)	33 (8.2)	4.755	5	0.447	0.091
	Low body image	30 (17.0)	37 (21.0)	45 (25.6)	19 (10.8)	27 (15.3)	18 (10.2)				
Olive oil	High body image	128 (31.7)	92 (22.8)	66 (16.3)	36 (8.9)	34 (8.4)	48 (11.9)	8.755	5	0.119	0.123
	Low body image	42 (23.9)	35 (19.9)	30 (17.0)	20 (11.4)	26 (14.8)	23 (13.1)				
Bread	High body image	298 (73.8)	46 (11.3)	28 (6.9)	12 (3.0)	3 (0.7)	17 (4.2)	7.017	6	0.319	0.110
	Low body image	119 (67.6)	24 (13.6)	17 (9.7)	5 (2.8)	5 (2.8)	6 (3.4)				
Pasta, rice	High body image	72 (17.8)	158 (39.1)	138 (34.2)	18 (4.5)	13 (3.2)	5 (1.2)	4.540	5	0.475	0.088
	Low body image	21 (11.9)	72 (40.9)	63 (35.8)	11 (6.3)	5 (2.8)	4 (2.3)				
Tea	High body image	326 (80.7)	38 (9.4)	23 (5.7)	6 (1.5)	2 (0.5)	9 (2.2)	2.377	5	0.795	0.064
	Low body image	134 (76.1)	21 (11.9)	12 (6.8)	2 (1.1)	2(1.1)	5 (2.8)				
Coffee	High body image	167 (41.3)	103 (25.5)	66 (16.3)	34 (8.4)	17 (4.2)	17 (4.2)	6.223	5	0.285	0.104
	Low body image	62 (35.2)	45 (25.6)	43 (24.4)	11 (6.3)	7 (4.0)	8 (4.5)				
Soda	High body image	39 (9.7)	52 (12.9)	97 (24.0)	73 (18.1)	63 (15.6)	80 (19.8)	3.898	5	0.564	0.082
	Low body image	10 (5.7)	27 (15.3)	43 (24.4)	28 (15.9)	27 (15.3)	41 (23.3)				

^a^χ² test

## Discussion

This study provides insight into the relationship between body image perception and health-related behaviors and beliefs regarding popular diets among female university students aged 18–33 years in Türkiye. Although previous research has associated body dissatisfaction with unhealthy weight control practices, evidence regarding its role in shaping awareness and engagement with popular diets remains limited [42–44].

The reason for limiting the sample to women was that, according to the literature, women generally exhibit higher levels of body dissatisfaction compared to men [45–47]. In this study, the relationship between body image and sociodemographic characteristics among university students was examined, and it was found that only body weight was significantly associated with body image scores (Table 1). This result is consistent with previous studies suggesting an association between body weight and body image perception. For example, Abu Alwafa and Badrasawi [48] reported a strong effect of body mass index on body image and body dissatisfaction in their study conducted among university students. Similarly, other studies have shown that a higher BMI is significantly associated with greater body dissatisfaction in both women and men [49, 50]. In a study conducted on university students, it was found that young adults from 22 countries who participated in the survey were particularly at risk because they perceived themselves as overweight despite having actual BMI values within the normal range [51]. This situation may represent a public health concern, as it has been associated with maladaptive weight control behaviors in previous studies. Furthermore, university students at the highest risk for body dysmorphic disorder were observed to have a thinner body image [52]. Numerous studies have reported that socioeconomic factors such as monthly expenditure and income status are associated with body image [53–55]. In the literature, regardless of body weight, the prevalence of low body image has been found to be higher among women with higher income levels [56, 57]. Similarly, in this study, some variation in body image scores was observed according to monthly and food expenditure categories; however, these differences did not reach statistical significance.

Table 2 shows that body image dissatisfaction is higher among individuals with chronic diseases. Similarly, a study conducted in Turkey with 3,153 participants reported a high prevalence of body dissatisfaction among individuals with chronic illnesses [47]. This finding may reflect perceptions regarding the impact of chronic illness on physical functioning and appearance [57]. In the current study, a relationship was found between body image and chronic illness types (*p* < 0.05) (Table 2). Metabolic and gastrointestinal conditions, in particular, anemia, gastritis/ulcers, and thyroid diseases, were observed at significant rates in both body image groups. Thyroid diseases and gastritis/ulcers were more common in the high body image group, while anemia was slightly more common in the low body image group. Anemia has been associated with low body image and self-esteem due to its chronic fatigue and physical appearance effects [58]. Thyroid diseases, particularly due to their effects on weight control, may contribute to low body image in individuals [59]. The observed differences in the distribution of chronic disease types between body image groups should be interpreted cautiously, as the underlying factors contributing to these patterns were not directly assessed in the present study. In this study, meal skipping behavior was found to be significantly associated with body image level (Table 2). Among individuals with high body image, the proportion of those who do not skip meals was higher, whereas a notably higher proportion of meal skipping was observed among individuals with low body image. These findings are consistent with the literature [47, 60]. This association may reflect a co-occurrence of low body image and engagement in weight control–oriented eating behaviors [61].

In the current study, a negative correlation was found between total body image scores and both skipping meals and BMI, while a positive correlation was observed with daily water intake (Table 3). No direct research has been found in the literature examining the relationship between daily water intake and the BIS; however, some studies have reported that higher water consumption is associated with healthier body composition and lower body fat [62, 63]. This association may be interpreted as an indirect relationship between health-related behaviors and body image perception. However, the observed correlations were generally weak in magnitude and should therefore be interpreted cautiously in terms of practical significance.

The strict beauty standards assigned to women’s bodies by society have been associated with body dissatisfaction, which is higher in women than in men [64]. Such societal pressures have been associated with increased interest in popular diets aimed at rapid weight loss [65]. When examining the association between body image level and popular diets, only nominal differences were observed for awareness of the monotrophic diet and practice of detox diets; however, these associations did not remain statistically significant after Bonferroni correction. Furthermore, the observed effect sizes were generally small and should therefore be interpreted with caution in terms of practical relevance. Overall, no robust associations were identified between body image perception and awareness, practice, or perceived healthiness of popular diets in this sample (Table 4). After controlling for multiple comparisons, no robust associations were observed between popular diet awareness and body image perception. No significant differences were observed for the other diet types (Table 4). Although these findings should be interpreted cautiously, the nominal differences observed for detox diet practices and monotrophic diet awareness may reflect greater interest in rapid weight-loss approaches among individuals with lower body image satisfaction. Recent studies indicate that concerns about appearance are associated with unhealthy eating behaviors and that body dissatisfaction is a global problem [66–68]. Ideal body perceptions for women are shaped through figures presented in fashion magazines, movies, and advertisements [69]. Accordingly, femininity and attractiveness for women are often equated with being “extremely thin” [70]. Studies conducted in Turkey and other countries support this view [68, 71]. Therefore, in this study, the greater awareness of diets aimed at rapid weight loss among individuals with low body image may reflect the widespread role of media exposure in shaping dietary awareness, rather than a direct influence of body image dissatisfaction. Data collection overlapped with early pandemic period; changes in routine and media exposure may have influenced diet-related awareness and behaviors. When asked where they heard about these diets, 70.7% of women reported media as the source (Table 5). In addition, unmeasured factors such as social media engagement, psychological distress, peer influence, and prior dieting experiences may also have contributed to the observed patterns and should be considered in future research.

Individuals with high body satisfaction are generally reported to have healthier eating habits, consuming products such as fruits, vegetables, and dairy more frequently [72, 73]. In the study conducted by Hernández Camacho et al. [74], a relationship between body image and fast food and vegetable consumption was reported; however, it was not clearly specified whether body image influences fast food consumption or vice versa. Furthermore, another study demonstrated that individuals who perceive their body weight to be higher than it actually is tend to preferentially select certain food groups [75]. However, in our study, no significant relationship was found between body image and any of the food consumption groups (Table 6). Similar findings have been reported in other studies [75, 76].

## Conclusion

This study examined the associations between body image perception, eating habits, health indicators, and attitudes toward popular diets among female university students. Higher body image scores were associated with healthier eating-related behaviors, including more regular meal consumption, higher water intake, and less frequent meal skipping. In contrast, no statistically significant associations were observed between body image perception and awareness, adoption, or perceived healthiness of popular diets, nor with food consumption frequency. Within the limitations of the current study design and sample, no strong associations were observed between body image perception and popular diet-related attitudes or behaviors. These findings suggest that while body image may be associated with certain health-related behaviors, no strong associations were observed between body image perception and awareness, adoption, or perceived healthiness of popular diets within the limitations of the current study design and sample. Future research and health promotion efforts may benefit from adopting a multidimensional perspective that considers sociocultural, psychological, and environmental influences on body image and dietary behaviors.

### Strengths and limitations

This study contributes to the existing literature by examining the relationship between body image perception and awareness of popular diets among female university students in Türkiye, a population in which body dissatisfaction is highly prevalent. The relatively large sample size (*n* = 580) strengthens the statistical robustness of the analyses and supports the reliability of the findings.

Several limitations should be acknowledged. The cross-sectional design precludes causal inferences between body image perception and dietary behaviors. In addition, the inclusion of only female university students limits the generalizability of the results to other populations. Another important consideration is that the study population consisted exclusively of female students enrolled in health-related departments. These students may have relatively higher baseline nutrition knowledge and greater exposure to health-related information compared with the general population. This relative homogeneity may have reduced variability in awareness and perceptions regarding popular diets, potentially contributing to a ceiling effect and limiting the ability to detect stronger associations. Therefore, the findings may not be generalizable to broader student populations or non-health-related groups.The reliance on self-reported data may have introduced recall or reporting bias. Importantly, key potential confounders such as social media use intensity, peer influence, prior dieting history, and psychological distress (e.g., anxiety or depressive symptoms) were not assessed. These factors may partially explain the observed associations between body image perception and diet-related awareness or practices. The use of dichotomous (yes/no) response categories may have limited the ability to capture the complexity, frequency, or intensity of participants’ perceptions and experiences regarding popular diets. In addition, the large number of statistical comparisons performed in the study may have increased the risk of both type I and type II errors. Although Bonferroni correction was applied to reduce the likelihood of false-positive findings, this conservative approach may also have reduced statistical sensitivity and increased the possibility of overlooking weak but potentially meaningful associations. Data were collected between February and June 2020, coinciding with the onset of the COVID-19 pandemic. Changes in daily routines and increased media exposure during this period may have influenced participants’ dietary behaviors and body image perceptions. Therefore, the findings should be interpreted within this contextual framework.

## Supplementary Information

Supplementary Material 1.

## Data Availability

The datasets generated during and/or analyzed during the current study are available from the corresponding author on a reasonable request.

## Abbreviations

BIS: Body image scale
BMI: Body mass index
