# Supplementary material for: Assessment of the relationship between awareness of popular diets and body image among young women: a cross-sectional study
Source: J Health Popul Nutr. 2026 Jul 7;45:211. doi: 10.1186/s41043-026-01388-6 (PMC13621742; doi:10.1186/s41043-026-01388-6)
Supplement: Supplementary file 1 [file 41043_2026_1388_MOESM1_ESM.docx]

**Supplementary Table S1.** Multivariable logistic regression analysis of factors associated with body image perception among female university students

| **Variable** | **Adjusted OR** | **95% CI** | **p value** |
| --- | --- | --- | --- |
| **BMI** | 0.963 | 0.918–1.011 | 0.133 |
| **Meal skipping (Yes vs. No)** | 0.469 | 0.285–0.772 | **0.003*** |
| **Monthly food expenditure** |  |  | 0.744 |
| 2000-4000 TL vs. 0-2000 TL | 1.213 | 0.513–2.870 | 0.660 |
| >4000 TL vs. 0-2000 TL | 1.354 | 0.581–3.156 | 0.483 |
| **Monthly expenditure** |  |  | 0.673 |
| 4000-8000 TL vs. 0-4000 TL | 0.982 | 0.447–2.159 | 0.965 |
| >8000 TL vs. 0-4000 TL | 0.827 | 0.400–1.712 | 0.609 |
| **Sources of diet-related information** |  |  | 0.457 |
| Media vs. reference category | 0.649 | 0.132–3.190 | 0.595 |
| Peers/social environment vs. reference category | 0.492 | 0.096–2.524 | 0.395 |
| Dietitian/health professionals vs. reference category | 0.868 | 0.157–4.793 | 0.871 |
| Presence of chronic disease | 1.206 | 1.026-1.419 | **0.023*** |

Abbreviations: OR, odds ratio; CI, confidence interval. The regression model demonstrated acceptable goodness-of-fit according to the Hosmer-Lemeshow test (χ²=3.750, df=8, p=0.879). *p<0.05.
